# Multimodal imaging diagnosis of pancreatic Castleman disease with abdominal and retroperitoneal lymphadenopathy: a case report and literature review

**DOI:** 10.3389/fonc.2024.1502878

**Published:** 2024-11-27

**Authors:** Chunchun Jin, Meifang Deng, Jing Zhang, Jianghao Lu, Yanli Hao

**Affiliations:** Department of Ultrasound, The First Affiliated Hospital of Shenzhen University Health Science Center, Shenzhen Second People’s Hospital, Shenzhen, China

**Keywords:** Castleman disease, pancreatic solid pseudopapillary tumors, pancreatic neuroendocrine tumors, diagnoses, case report

## Abstract

**Introduction:**

Castleman’s disease (CD) represents a rare polyclonal lymphoproliferative disorder characterized by atypical lymph node hyperplasia, the precise etiology of which remains undefined. Pancreatic involvement of CD is particularly uncommon and often misdiagnosed due to its nonspecific clinical features, making it difficult to distinguish from tumors with abundant blood supply such as solid pseudopapillary tumors and neuroendocrine tumors. Multimodal imaging plays a crucial role in diagnosing pancreatic CD and determining the extent of lymph node involvement. Surgical resection is the preferred treatment for unicentric CD (UCD). Regular follow-up and monitoring are essential for optimal patient management and treatment outcomes.

**Case Presentation:**

A 26-year-old female presented to our hospital with a pancreatic mass detected during a routine health examination. Physical and laboratory examinations were unremarkable. Conventional ultrasound revealed a hypoechoic, oval-shaped mass measuring approximately 34×31 mm in the pancreatic head, with clear boundaries and heterogeneous internal echoes, but no significant blood flow signals. Contrast-enhanced ultrasound showed hyperenhancement of the pancreatic head mass, raising suspicion for a solid pseudopapillary tumor or other entities. To clarify the diagnosis, Contrast enhanced CT, MRI, and FDG PET-CT were performed. Both Contrast enhanced CT and MRI demonstrated hyperenhancement of the lesion, and PET-CT showed hypermetabolism in the pancreatic head mass, along with multiple enlarged lymph nodes in the abdominal and retroperitoneal regions. These findings suggested a lymph node origin for the pancreatic head mass. Surgical resection was performed, and pathological examination confirmed UCD. The patient has been followed for 44 months without recurrence.

**Conclusion:**

This case illustrates the utility of multimodal imaging in diagnosing pancreatic CD. In cases of benign pancreatic lesions with enlarged abdominal and retroperitoneal lymph nodes, CD should be considered.

## Introduction

Castleman’s disease (CD), a distinctive lymphoproliferative disorder, demonstrates an estimated incidence of 21 cases per million population (1). The disease exhibits no significant gender predilection and manifests across all age groups. The precise etiopathogenesis of CD remains to be fully elucidated. CD is primarily classified into two subtypes: unicentric CD (UCD), characterized by single or multiple lymph node involvement confined to a solitary anatomic region, typically presenting with an indolent clinical course; and multicentric CD (MCD), distinguished by lymphadenopathy involving multiple anatomic regions, frequently accompanied by systemic manifestations including pyrexia, nocturnal diaphoresis, and fatigue (2, 3). While lymph nodes represent the predominant sites of involvement in CD, pancreatic manifestation is an uncommon presentation. The diagnostic challenge of pancreatic CD is compounded by its low incidence and non-specific clinical features, frequently leading to misdiagnosis. Implementation of comprehensive and standardized diagnostic algorithms and therapeutic protocols is fundamental for optimal patient management. This investigation presents a case of pancreatic CD diagnosed through multimodal imaging and systematically reviews the enhancement patterns of pancreatic CD documented in the existing literature. The primary objective is to establish imaging-based diagnostic criteria that will facilitate accurate identification of pancreatic CD in clinical practice.

## Case presentation

A 26-year-old female patient presented to our hospital following the incidental discovery of a pancreatic mass during a routine physical examination conducted 10 days prior. The patient exhibited no clinical symptoms, and physical examination upon admission revealed no positive findings. Laboratory tests showed no significant abnormalities.

**Figure 1:**
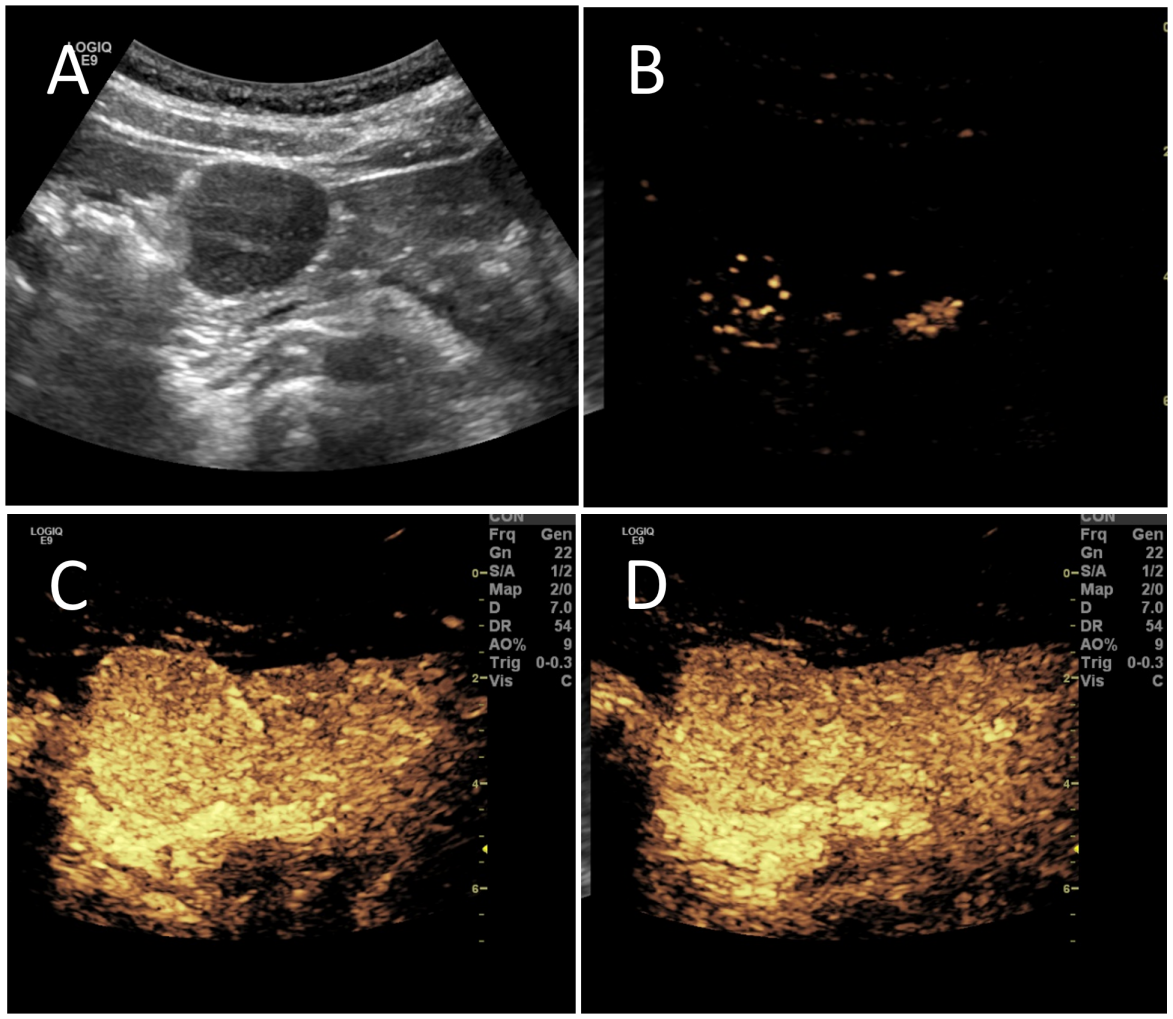
The patient underwent conventional ultrasound and contrast-enhanced ultrasound (CEUS). **(A)** Conventional ultrasound revealed a hypoechoic mass in the pancreatic head region, measuring approximately 34×31mm. The mass was oval-shaped with clear boundaries and heterogeneous internal echoes, featuring a few “fissure-like” anechoic areas and slightly enhanced posterior echoes. **(B)** CEUS showed enhancement of the mass beginning at 11 seconds. **(C)** The enhancement peaked at 19 seconds, displaying hyperenhancement. **(D)** At 34 seconds, the enhancement began to decline, showing slight hyperenhancement with peripheral ring enhancement.

**Figure 2:**
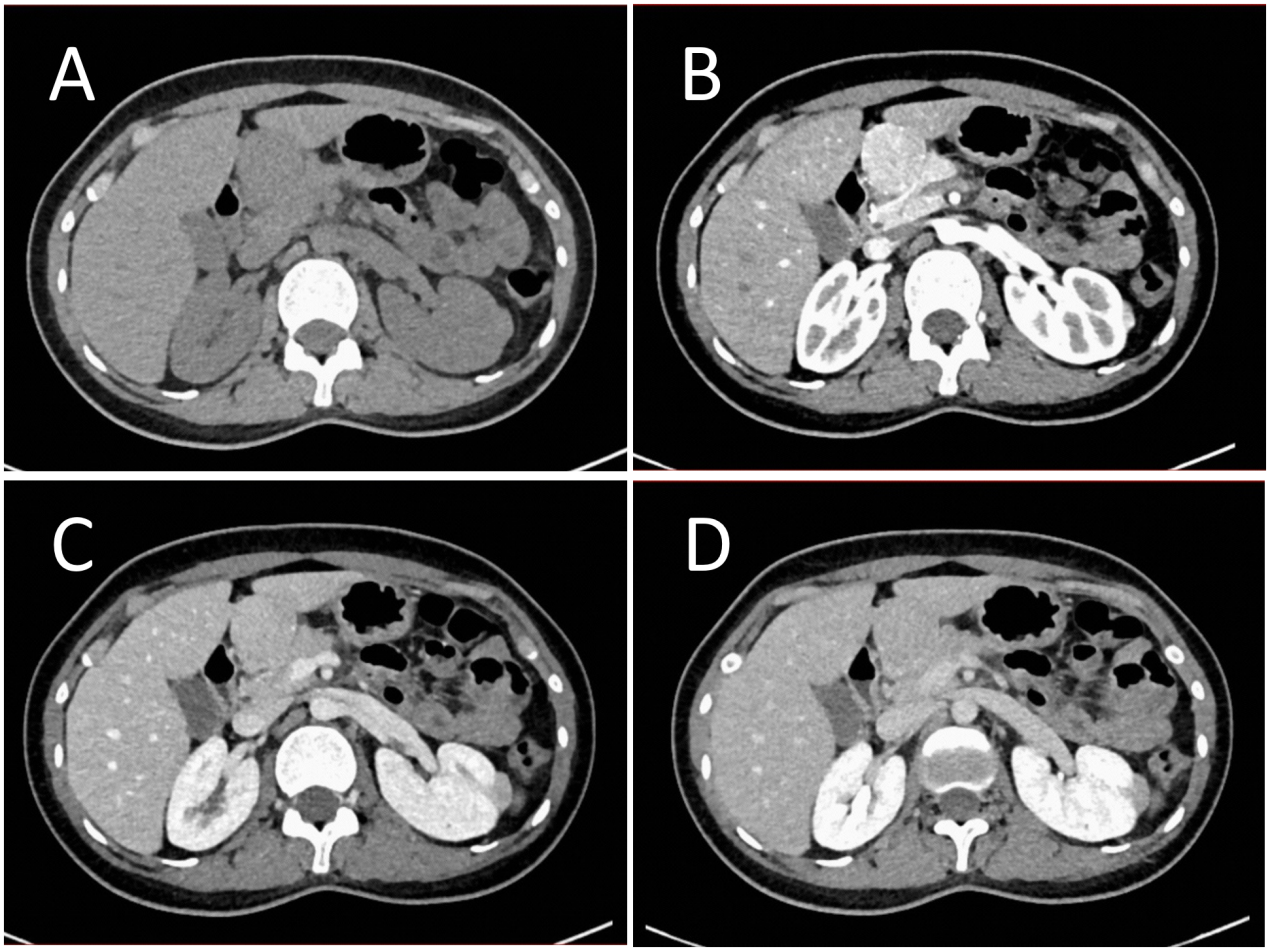
Contrast enhanced CT evaluation of the lesion. **(A)** CT imaging showed a round-shaped lesion in the pancreatic head region with a CT value of approximately 50 HU, clear boundaries, and slight compression narrowing of the adjacent descending duodenum. **(B)** Enhanced scanning revealed the lesion with a CT value of approximately 145 HU during the arterial phase, showing significant homogeneous enhancement. **(C)** During the pancreatic parenchymal phase, the lesion had a CT value of approximately 143 HU, indicating slight hyperenhancement. **(D)** In the portal venous phase, the lesion had a CT value of approximately 117 HU, also showing slight hyperenhancement.

**Figure 3:**
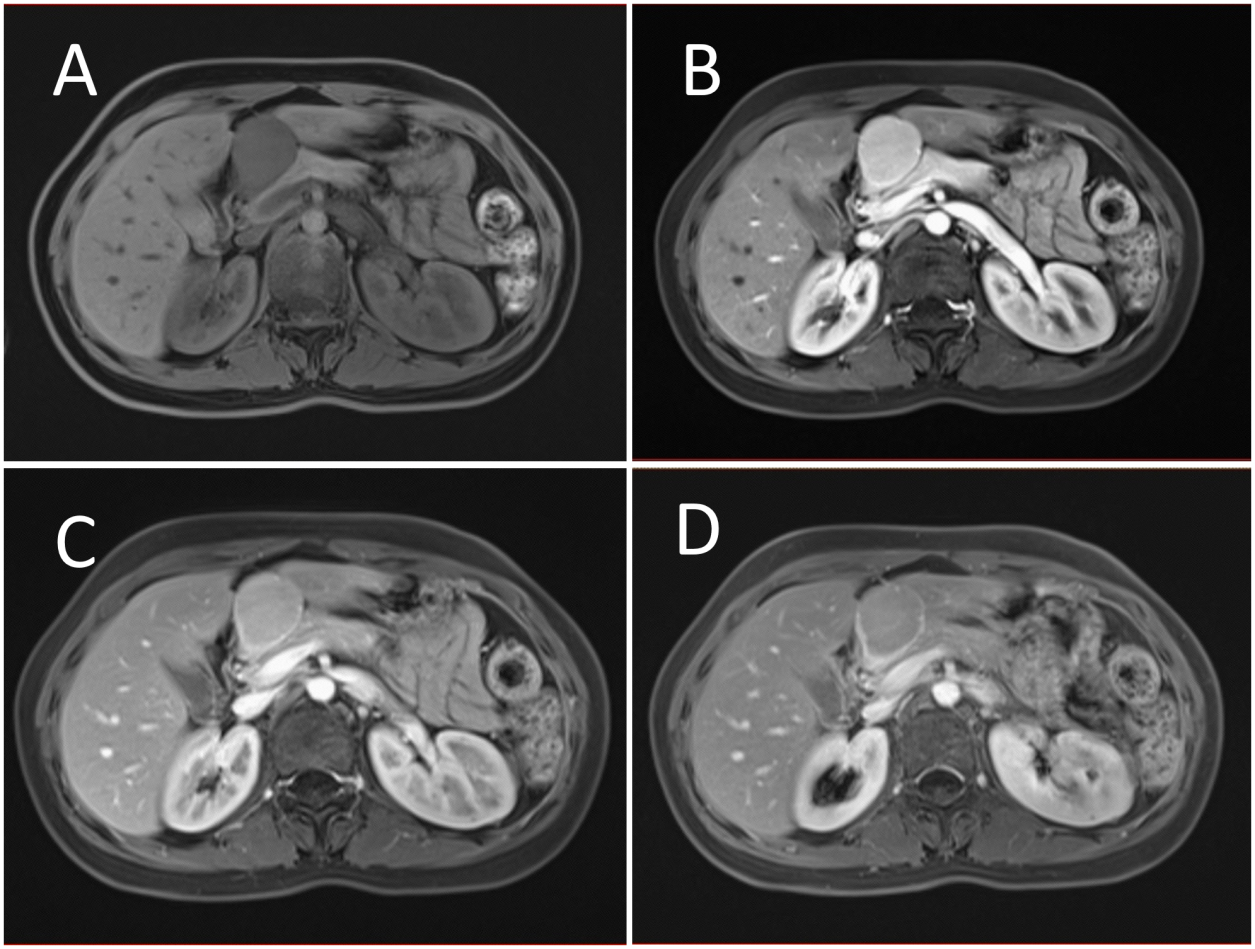
Contrast enhanced MRI evaluation of the lesion. **(A)** MRI revealed a solid abnormal signal mass in the pancreatic head region, with a regular shape and clear boundaries. **(B)** Enhanced scanning showed significant homogeneous enhancement during the arterial phase. **(C)** The lesion displayed slight hyperenhancement during the parenchymal phase. **(D)** During the portal venous phase, the lesion also showed slight hyperenhancement. Nodular mild enhancement was observed within the lesion, with peripheral ring enhancement, and the lesion’s margins remained clear post-enhancement.

**Figure 4:**
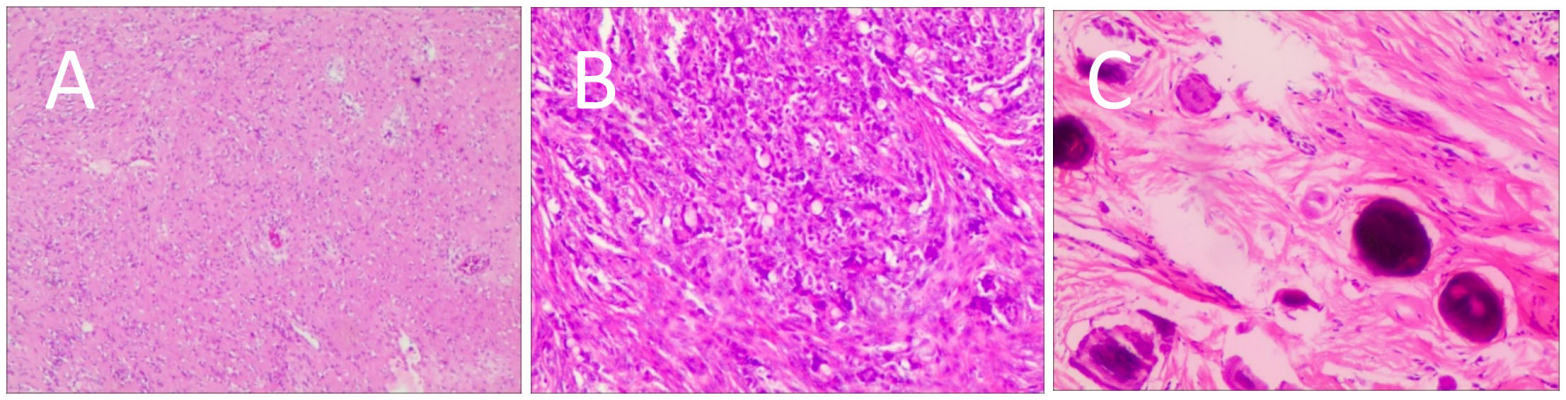
Postoperative histological features. **(A)** Most lymphatic sinuses in the lymph nodes were absent. **(B)** Hyperplastic follicles were evenly distributed throughout the lymph node. **(C)** Small blood vessels were observed growing into the lymphatic follicles, with thymus-like changes in the germinal center endothelial cells. The mantle zone lymphocytes were broadened, sometimes displaying an “onion-skin” arrangement. There was an increase in small blood vessels between follicles, with hyaline changes, and proliferation of some plasma cells and immunoblasts.

B-mode ultrasound identified a 34×31mm hypoechoic mass in the head of the pancreas, with distinct boundaries and heterogeneous internal echoes. Color Doppler imaging showed linear blood flow signals within the mass. The body and tail of the pancreas appeared normal, and the main pancreatic duct was not dilated (**Figure 1A**). To clarify the diagnosis, the patient underwent contrast-enhanced ultrasound (CEUS) examination. CEUS showed the mass exhibited initial enhancement at 11 seconds, peaking at 19 seconds, and began washing out at 34 seconds, displaying slight hyperenhancement with peripheral ring enhancement (**Figures 1B–D**). CEUS suggested a solid pseudopapillary tumor or other potential diagnoses. The lesion was further evaluated by computed tomography (CT) and magnetic resonance imaging (MRI). CT imaging revealed a round lesion with clear boundaries in the head of pancreas head, adjacent to the descending part of the duodenum, causing slight compression and narrowing of the lumen (**Figure 2A**). Contrast-enhanced CT showed the lesion with homogeneous hyperenhancement during the arterial phase and slight hyperenhancement during the pancreatic parenchymal and portal venous phases (**Figures 2B–D**). Multiple enlarged lymph nodes were observed in the abdomen and retroperitoneum. CT suggested a lesion possibly originating from lymph nodes or other sources. MRI demonstrated a solid mass with abnormal signal intensity in the pancreatic head (**Figure 3A**). Contrast-enhanced MRI revealed uniform hyperenhancement during the arterial phase and slight hyperenhancement during the parenchymal and portal venous phases. Nodular mild enhancement was observed within the lesion, with peripheral ring enhancement, and the lesion’s margins remained clear post-enhancement (**Figures 3B–D**). MRI suggested a solid pseudopapillary tumor or a lymphatic origin. To comprehensively assess the disease involvement of other parts of the body, FDG PET-CT examination was performed. The PET-CT examination conducted in the hospital outside revealed a slightly hypodense mass between the pancreatic head and the liver, showed hypermetabolism in the mass. Additionally, multiple enlarged lymph nodes were observed around the abdominal aorta in the abdominal and retroperitoneal regions, with no hypermetabolism. Preoperative evaluations of other organs showed no significant abnormalities. The differential diagnosis for the mass in the pancreatic head region includes a lymph node-derived space-occupying lesion, a solid pseudopapillary tumor, or other potential diseases. Due to the patient’s refusal of a biopsy, laparoscopic excision of the pancreatic head mass was performed. The mass was gray-brown, solid, and moderately firm. Histopathological Features: Most lymphatic sinuses in the lymph nodes were absent (**Figure 4A**). Hyperplastic follicles were evenly distributed throughout the lymph node (**Figure 4B**). Small blood vessels were observed growing into the lymphatic follicles, with thymus-like changes in the germinal center endothelial cells. The mantle zone lymphocytes were broadened, sometimes displaying an “onion-skin” arrangement. There was an increase in small blood vessels between follicles, with hyaline changes, and proliferation of some plasma cells and immunoblasts (**Figure 4C**). Immunohistochemistry results showed CD5 (–), CyclinD1(-), Bcl-2(+, germinal center -), Bcl-6(-), CD20(+), CD3(+), CD21 (expanded FDC network +), CD23 (expanded FDC network +), Ki-67 (5% +), Kappa (focal +), Lambda (scattered +), MUM-1(-), TdT(-), TPO(-), CD38 (small focal +). The pathology report suggested pancreatic Castleman disease, hyaline vascular type (HV-CD).

The patient recovered well postoperatively, with no tumor recurrence observed over a 44-month follow-up period.

## Discussion

While CD most commonly affects lymph nodes, its occurrence in the pancreas is rare. Although reports on CD are increasing, it remains a rare disease (4). The 2020 consensus guidelines from the CD Collaborative Network emphasize the importance of individualized treatment (5). Therefore, individualized diagnosis is crucial for patients suspected of having CD.

According to the 2020 consensus guidelines established by the CD Collaborative Network, the diagnostic algorithm for CD encompasses a comprehensive evaluation protocol, integrating clinical assessment, Laboratory tests, imaging examination, and histopathological examination (5). In this case, the patient was incidentally diagnosed during a routine physical examination and displayed no obvious clinical symptoms. Ultrasound revealed a hypoechoic mass in the pancreatic head with a regular shape and clear boundaries. CEUS showed the lesion with hyperenhancement and clear post-enhancement margins. Pancreatic cancer, typically a hypovascular tumor with irregular shapes and unclear boundaries, was excluded based on clinical and laboratory findings. CEUS suggested a high likelihood of a benign lesion, considering possibilities such as pancreatic neuroendocrine tumors, solid pseudopapillary tumors, or other benign tumors. Given the patient’s young age and gender, CEUS favored a diagnosis of a solid pseudopapillary tumor. To further assess pancreatic involvement and evaluate abdominal organs comprehensively, CT and MRI were performed. Contrast enhanced CT and MRI showed the lesion with homogeneous hyperenhancement during the arterial phase and slight hyperenhancement during the pancreatic parenchymal and portal venous phases, indicating a rich blood supply. The slight compression and narrowing of the duodenal lumen adjacent to the lesion were likely due to the mass’s location in the pancreatic head. The enhancement margins of pancreatic CD was clear and absence of signs of invasion into adjacent tissues, indicating a benign mass. Differential diagnosis of this lesion primarily includes solid pseudopapillary neoplasm (SPN) and pancreatic neuroendocrine tumor (pNET). SPN typically presents as a solid or cystic and solid mass with a characteristic thick fibrous capsule. On contrast-enhanced imaging, SPN demonstrates hypoenhancement during both arterial and pancreatic parenchymal phases, with prominent enhancement of the peripheral fibrous capsule (6). Functional pNETs are typically associated with specific clinical syndromes. While non-functional pNETs are rare in clinical practice, they often present with a well-defined capsule and may exhibit hemorrhagic or cystic degeneration. On contrast-enhanced imaging, pNETs characteristically demonstrate hyperenhancement or isoenhancement during the arterial phase, followed by hypoenhancement during the pancreatic parenchymal phase (6). Additionally, multiple enlarged lymph nodes were observed in the abdomen and retroperitoneum in CT and MRI FDG PET-CT scan demonstrated hypermetabolism in the mass located in the pancreatic head region, along with multiple enlarged lymph nodes surrounding the abdominal aorta with no hypermetabolism. which needs to be differentiated from lymphoma. Lymphoma typically presents with systemic manifestations, including pyrexia, nocturnal diaphoresis, and unintentional weight loss etc. Lymphoma characteristically demonstrates hypermetabolism in PET-CT (7). The radiological and clinical findings suggest that the pancreatic head mass might meet the diagnostic characteristics of CD.

A review of previously published literature on the enhancement patterns of pancreatic CD was shown in **Table 1**. Dev et al. (8–10) and our study both exhibited hyperenhancement during the arterial phase. Ferreira Junior et al. (11) reported a pancreatic head lesion with homogeneous enhancement on MRI, indicating a rich blood supply in pancreatic CD. In this case, the combination of ultrasound and CEUS effectively localized the lesion and assessed its size, morphology, and perfusion, aiding in the differentiation between benign and malignant lesions. The misdiagnosis by CEUS may have been due to the lesion’s rich blood supply, initially suggesting a benign pancreatic tumor. However, CEUS scanning range was limited for the abdomen and retroperitoneum, coupled with the rarity of CD, meant that this rare disease was not initially considered. CT, MRI and FDG PET-CT with their broader scanning range, clarified the lesion’s enhancement pattern, its relationship with surrounding tissues, and the status of abdominal and retroperitoneal lymph nodes, facilitating a comprehensive disease assessment. For benign pancreatic lesions with accompanying abdominal and retroperitoneal lymphadenopathy, CD should be considered. Through a comprehensive review of the clinical symptoms, laboratory findings, and multimodal imaging features of pancreatic CD with abdominal/retroperitoneal lymphadenopathy, which is helpful to understand the imaging characteristics of pancreatic CD, evaluate the relationship between the lesion and surrounding tissues and provide imaging-based evidence for establishing comprehensive diagnostic criteria in future guidelines.

**Table 1:** A review of published literature on the enhancement patterns of pancreatic CD was shown.

Author	Year	Country	Sex	Age	Symptoms	Location	Maximum diameter	Pancreatic/Bile duct dilatation	Duodenum compression	Enhanced mode	Lympha-denectasis	Follow up	Surgery
Ferreira Junior et al. (11)	2019	Brazil	Woman	34	Intermittent abdominal pain	Head of the pancreas	4.0 cm	Bile duct dilatation	No	CEMRI: homogeneous hypoenhancement	No	12 m	Excision
Dev et al. (8)	2023	Nepal	Woman	46	Incidental	Body of the pancreas	4.1 cm	No	No	CECT: homogeneous hyperenhancement	No	2 m	Excision
Liu et al. (10)	2022	China	Woman	28	Incidental	Neck of the pancreas	3.0 cm	No	No	CECT: homogeneous hyperenhancement	No	6 m	Excision
Zhai et al. (9)	2022	China	Woman	44	Incidental	Body of the pancreas	3.2 cm	No	No	CEUS: homogeneous hyperenhancement. CECT and CEMRI: hyperenhancement in the arterial phase and slightly hyperenhancement in the other phases	Yes	No	Excision
David et al. (13)	2022	Australia	Woman	61	Vomiting, diarrhoea and abdominal discomfort	Body of the pancreas	2.8cm	No	No	FDG PET-CT: intense avidity	No	12 m	Excision
This study	2024	China	Woman	26	Incidental	Head of the pancreas	3.4 cm	No	Yes	CEUS: homogeneous hyperenhancement. CECT and CEMRI: hyperenhancement in the arterial phase and slightly hyperenhancement in the other phase FDG PET-CT: intense avidity	Yes	44 m	Excision

CECT, Contrast-enhanced Computed Tomography; CEMRI, Contrast-enhanced Magnetic Resonance Iimaging; m, mohth.

For patients with UCD, surgical resection is the preferred treatment (12). In this case, the patient recovered well postoperatively. Follow-up examinations at 1 month, 3 months, 6 months, 1 year, 2 years, and 3 years post-surgery have been conducted, with no recurrence observed over a 44-month follow-up period.

## Conclusion

Pancreatic CD is very rare. This case of pancreatic CD showed hyperenhancement on CEUS, enhanced CT and MRI, which was difficult to distinguish from SPN and pNET. The combination of clinical manifestations and enhancement of the lesion capsule was helpful for the differential diagnosis of the lesion. When the lesion is accompanied by abdominal/retroperitoneal lymph node enlargement, it is difficult to differentiate the lesion from lymphoma. PET-CT was helpful for the differential diagnosis through the radioactive uptake of the lesion. Multimodal imaging is essential for a comprehensive assessment of pancreatic CD, enhancing diagnostic accuracy.

## Data Availability

The original contributions presented in the study are included in the article/supplementary material. Further inquiries can be directed to the corresponding author.
